# High endothelial venules in intracranial germinomas: Implications for lymphocytes infiltration

**DOI:** 10.1002/cam4.5367

**Published:** 2022-10-19

**Authors:** Huiyuan Chen, Guilin Li, Yun Cui, Qi Zhang, Bo Li, Xing Liu

**Affiliations:** ^1^ Department of Neuropathology Beijing Neurosurgical Institute, Capital Medical University Beijing People's Republic of China; ^2^ Department of Ultrastructure Pathology Beijing Neurosurgical Institute, Capital Medical University Beijing People's Republic of China; ^3^ Department of Radiation Oncology Beijing Tiantan Hospital, Capital Medical University Beijing People's Republic of China

**Keywords:** gene ontology, germinoma, high endothelial venules (HEVs), peripheral node addressin (PNAd), tumor‐infiltrating lymphocytes, xCell

## Abstract

**Purpose:**

Reactive lymphocytes are substantial components of germinoma, which are believed to be related to the favorable prognosis of this intracranial tumor and better response to immunotherapy. However, the mechanisms managing the recruitment of lymphocytes are poorly understood. High endothelial venules (HEVs) are specialized blood vessels that play key roles in lymphocyte trafficking in Lymph nodes. These vessels are associated with lymphocyte infiltration in chronic inflammatory diseases and various malignant tumors, but their distribution and implications in germinoma are unknown. This study aimed to investigate the distribution and implications of HEVs in intracranial germinomas.

**Methods:**

We investigated the presence and distribution of HEVs in 42 germinomas by immunohistochemical staining of peripheral node addressin (PNAd) and transmission electron microscopic examination. The correlation of the densities of HEVs with the extent of T and B lymphocyte infiltration and several clinicopathological characteristics were also analyzed to determine whether HEVs are responsible for lymphocyte recruitment and their roles in anti‐tumor immunity in germinoma.

**Results:**

PNAd‐positive HEVs were detected in 31% (13/42) of germinomas, and their presence correlated with abundant infiltrating CD3+ T cells, CD20 + B cells and CD8+ cytotoxic T lymphocytes (*p* = 0.0410, 0.0023, and 0.0061, respectively). Higher HEVs density was also correlated with several clinicopathological parameters, which are recognized indicators for favorable prognosis in germinomas, including typical tumor location (*p* = 0.0093), lower tumor cell content (*p* = 0.0428), and younger age at diagnosis (*p* = 0.0121). Furthermore, bioinformatics analysis showed HEVs‐associated genes mainly enriched in immune‐related Gene Ontology terms, including innate immune response, inflammatory response, and B cell receptor signaling pathway. The xCell analysis revealed that germinomas with higher HEVs enrichment scores had increased levels of the immune score, microenvironment score, dendritic cells, CD8+ central memory T‐cells, CD4+ memory T‐cells, and B‐cells.

**Conclusions:**

Our findings indicate that HEVs could contribute to lymphocyte recruitment in germinomas, thus may serve as a predictor of favorable prognosis and better response to immunotherapy in this intracranial tumor.

## 1 INTRODUCTION

Germinoma is the most common germ cell tumor in the central nervous system, which principally affects children and adolescents. The incidence of this tumor in eastern Asia is more than triple that in Europe and the United States.^1^ Pure germinoma is extremely sensitive to radiotherapy and chemotherapy, thus being clinically curable. However, various side effects of these aggressive therapies, such as cognitive dysfunction, growth retardation, and secondary neoplasms, could seriously impair the life quality of young patients. Developing new therapeutic approaches are therefore needed.

Immunotherapy for several kinds of tumors has achieved great success in the last decade. While various strategies, such as adoptive lymphocyte transfers, vaccines, and checkpoint blockade, have little to no effect if there are insufficient lymphocytes in the tumor microenvironment. At the same time, reactive lymphocytes are substantial components besides large primordial germ cells in germinoma.^2^ These small inflammatory infiltrations can function as significant effector cells for immunotherapies. Besides, it is believed that these cells are toxic to the tumor cells and are significantly associated with a better prognosis in germinomas.^2^, ^3^, ^4^ Therefore, it is essential to understand the mechanisms of lymphocyte recruitment in this intracranial tumor.

High endothelial venules (HEVs) are specialized post‐capillary venules that efficiently recruit lymphocytes from blood circulation into lymph nodes. The distinctive features of HEVs include the cuboidal endothelial cells and positive expression of peripheral node addressin (PNAd) on the apical surface of these cells. These characteristics can distinguish HEVs from other types of vessels under light microscopic examination. Besides lymph nodes and other secondary lymphoid organs, blood vessels with HEV‐like characteristics can also present in various kinds of chronic inflammatory diseases, including rheumatoid arthritis,^5^, ^6^, ^7^ Crohn's disease,^8^ and ulcerative colitis.^6^, ^9^ Recently, HEV‐like vessels are induced in human malignancies of several origins, such as lung,^10^ colon,^11^ breast,^12^, ^13^ gastric^14^, ^15^ cancer, and melanomas,^16^ especially in testicular seminomas,^17^ the counterpart of intracranial germinoma. Furthermore, the density of HEV‐like vessels was strongly correlated with T and B lymphocyte infiltration in these tumors. Together, these findings suggest that HEVs may play a critical role in lymphocyte trafficking to tumors, therefore, are important for improving anti‐tumor immune response and enhancing immunotherapy efficiency.

This study aimed to investigate the distribution and implications of HEVs in intracranial germinomas. The presence of HEVs was analyzed using immunohistochemical staining, immunofluorescence staining, and transmission electron microscopic examination. The correlation between the density of HEVs with the extent of T‐ and B‐cell infiltration and several clinicopathological characteristics were also analyzed to determine whether HEVs are responsible for lymphocyte recruitment and their roles in anti‐tumor immunity in germinoma. Furthermore, we conducted a preliminary bioinformatic analysis on a series of transcriptome data to assess HEV‐related biological processes and enriched cell subsets.

## 2 MATERIALS AND METHODS

### 2.1 Patients and samples

We retrospectively identified 42 patients with pure intracranial germinoma who received surgical resection or stereotactic biopsy between 2016 and 2020 in the Department of Neurosurgery at Beijing Tiantan Hospital, China. The histopathological diagnoses were determined by routine evaluation of Formalin‐fixed paraffin‐embedded samples with hematoxylin and eosin (H&E) staining. All the 42 cases showing pure germinoma features without any other germ cell components were checked by at least two experienced neuropathologists. Formalin‐fixed paraffin‐embedded tissue blocks with adequate tumor tissue of these cases were obtained from the Department of Neuropathology. One patient's freshly resected tumor specimen was conducted for the transmission electron microscopic examination.

Clinical and pathological information, including age, gender, tumor volume, and tumor location, were collected and summarized in Table 1. The tumor volume was evaluated by measuring them in pre‐surgery magnetic resonance images. Tumor locations were classified into three groups: suprasellar compartment, pineal region, and other locations. Other locations in this study include basal ganglia, thalami, midbrain, cerebellum, and spinal cord.

**TABLE 1:** Clinicopathological characteristics of germinoma patients

	Number (%)
Ages(years)	
Median	16
Range	6–39
Tumor location	
Suprasellar	15 (35.7%)
Pineal region	15 (35.7%)
Other location	12 (28.6%)
Gender	
Female	14 (33.3%)
Male	28 (66.7%)
Volume (cm^3^)	
Mean	57
Range	1.7–491.4
HEV	
High	7 (16.7%)
Low	35 (83.3%)

Tumor cell content was determined by measuring the proportion of neoplastic elements, which were marked by immunohistochemical staining of organic cation/carnitine transporter4 (OCT4) among all kinds of cells in the tumor microenvironment. The measurement was conducted in the slides with the most abundant tumor cells.

### 2.2 Immunohistochemical and immunofluorescence staining

Immunohistochemistry and immunofluorescence were performed on 4‐μm thick sections from the tissue blocks. The primary antibodies utilized in this study were as follows: PNAd Carbohydrate Epitope (Clone MECA‐79, 1:40 dilution, BD Pharmingen, Becton); CD3 (clone LN10, pre‐diluted, ZSGB‐BIO), CD8 (clone SP16, pre‐diluted, ZSGB‐BIO), CD20 (clone L26, pre‐diluted, ZSGB‐BIO), and OCT4 (clone N1NK, pre‐diluted, ZSGB‐BIO). For Immunohistochemical staining, slides were incubated in an automated IHC Staining System (BOND‐III, Leica Biosystems Newcastle Ltd) and visualized by Bond Polymer Refine Detection Kit (DS9800, Leica Biosystems Newcastle Ltd). For immunofluorescence staining, slides were incubated with secondary antibodies and counterstained with DAPI.

### 2.3 Quantification of HEVs and lymphocyte subsets

For HEVs, we first counted the number of MECA‐79‐positive vessels in each tumor slide under a light microscope. Then we determined HEVs density by calculating them as counts per mm^2^ tumor area. In accord with the cutoff points for tumor HEVs density previously described,^12^ tumors with HEVs more than 0.20/mm^2^ were designated as HEV‐high germinomas. In contrast, those with HEVs less than 0.20/mm^2^ were defined as HEV‐low germinomas.

The amounts of CD3+ T cells, CD20+ B cells, and CD8+ cytotoxic T lymphocytes (CTLs) were determined by automatically counting these cells in five representative high‐power fields (HPF) with the most prominent tumor‐infiltrating lymphocytes (TILs). The counting was conducted using Image J Software (NIH).

### 2.4 Transmission electron microscopic examination

Fresh germinoma tissue (1 mm^3^) was fixed in 2.5% glutaraldehyde and 2% polyformaldehyde at 4°C for 2 hours. After rinsing with PBS, the tumor tissue was fixed in 1% osmium acid, followed by acetone dehydration, soaking, and epoxy resin embedding. One‐micrometer semi‐thin sections were then prepared and stained using the Azure‐methylene Blue Dye for localization analysis. Next, we prepared 80‐nm ultrathin sections and stained them with uranium dioxyacetate‐lead citrate. The ultrathin sections were observed by a Hitachi H‐7650 transmission electron microscope (Hitachi).

### 2.5 Statistical analysis

Statistical analyses were conducted using the GraphPad Prism 7 software (GraphPad Software) and SPSS (IBM). Differences in HEVs density among groups categorized by gender, age, and tumor location were analyzed using the chi‐square test. Correlations of HEVs density with tumor volume, tumor cell content, and the amount of infiltrating CD3+ T cells, CD20 + B cells, and CD8+ CTLs were calculated using the Mann–Whitney *U*‐test. Two‐sided *p* values less than 0.05 were considered statistically significant.

### 2.6 Bioinformatic analysis

The transcriptome data were obtained from the Gene Expression Omnibus Datasets (GSE19348).^18^ 13 central nervous system Germ cell tumors (GCT) cases with different histological subtypes were included in this Serie, of which six were pure germinoma. The enrichment of HEVs was evaluated using the Single‐Sample Gene Set Enrichment Analysis (ssGSEA). The HEVs‐associated gene set consisted of sialomucins‐related genes (CD34, PODXL, EMCN, CD300LG), enzymes‐related genes (CHST4, CHST2, FUT7, FUTt4, GCNT1, B3GNT3, and ST3GAL6),^19^ and CCL21, which was highly expressed in HECs.^20^ According to their median HEVs enrichment scores, the six germinomas were designated as the HEV‐high or HEV‐low group. The differentially expressed genes were identified using Student's *t*‐tests (*p* < 0.05, Fold change > 20%). Gene Ontology (GO) analysis was applied on the HEVs‐associated highly expressed genes. Meanwhile, the xCell algorithm was used to calculate the cell‐type enrichment scores.^21^ Cell types with enrichment scores >0 in more than 80% of cases were then subjected to correlation analyses.

## 3 RESULTS

### 3.1 Blood vessels with HEVs characteristics in germinomas

**FIGURE 1:**
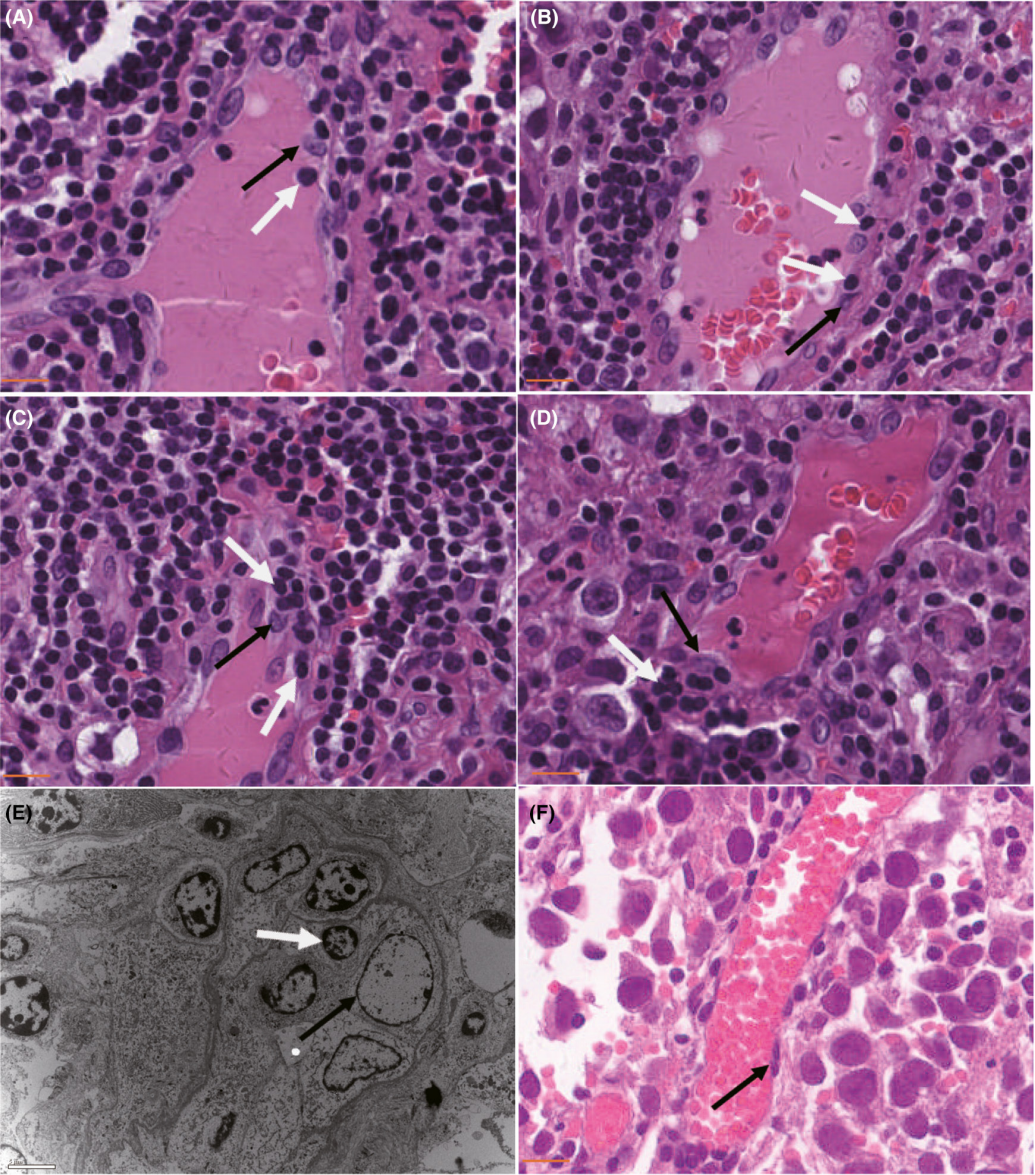
Representative histological morphology of HEVs. (A–D) The representative images of lymphocytes' extravasation process: The lymphocytes are arrested on the luminal surface of cuboidal endothelial cells (A), get in the endothelial lining of HEVs (B), arrive at the external side of endothelial cells (C), and finally cross the basal lamina (D). (E)Transmission electron micrography (TEM) showed lymphocytes squeezed between the endothelial cells. (F) Endothelial cells of non‐HEVs‐like vessels are flat. White arrows: lymphocytes. Black arrows: endothelial cells. The scale bar is 50 μM for H&E and 5 μM for TEM images.

**FIGURE 2:**
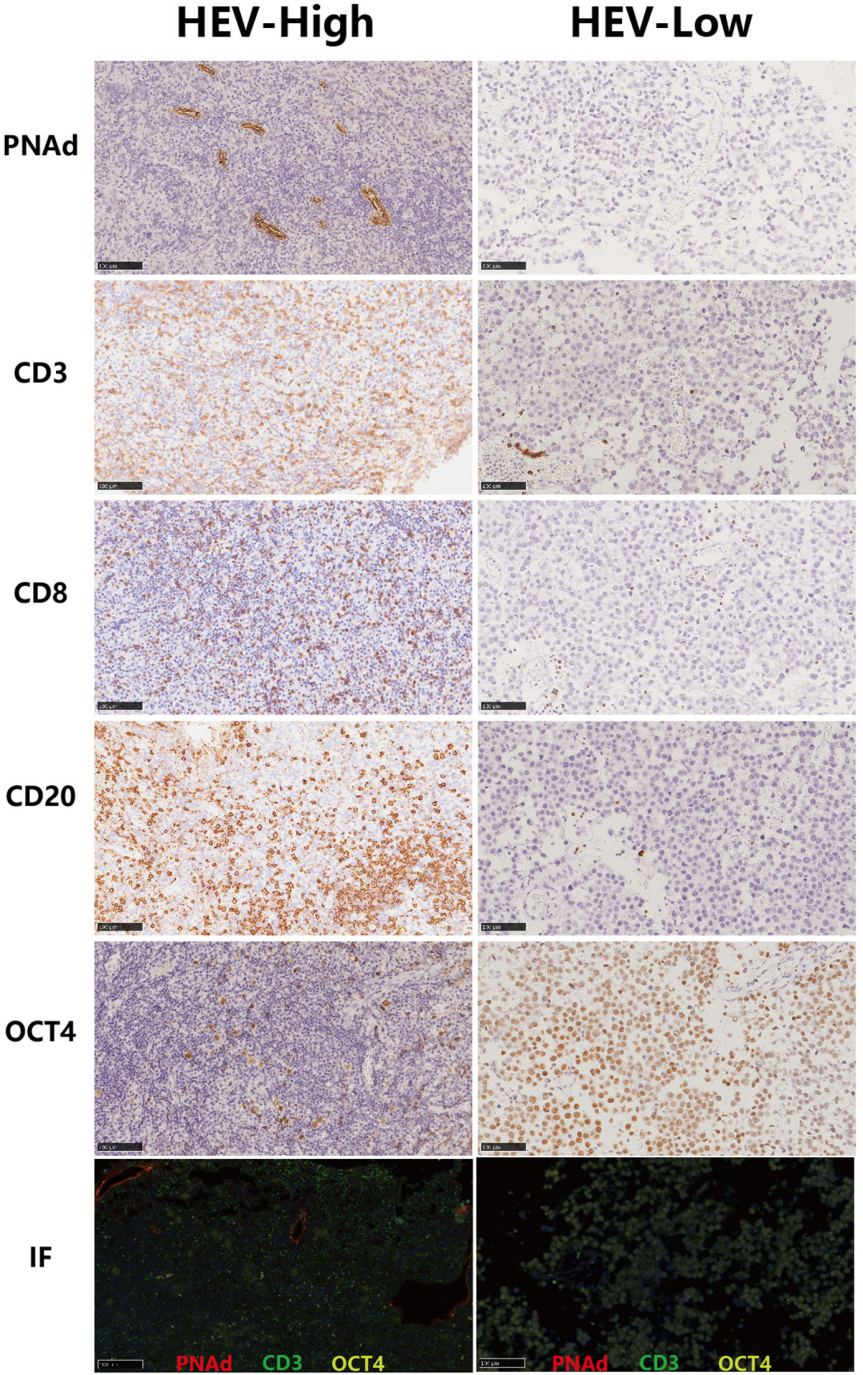
Distinct features on cellular components in HEV‐high or HEV‐low germinomas. Immunohistochemical (IHC) staining for PNAd showed HEVs, organic cation/carnitine transporter4 (OCT4) showed germinoma cells, CD3 and CD8 showed T cells, and CD20 showed B cells. Immunofluorescence (IF) staining showed that MECA‐79‐positive HEVs (red) are accompanied by CD3‐positive (green) T lymphocytes in germinoma. Scale bars were 100 μm for IHC and IF staining images.

In total, 42 cases of pure intracranial germinoma patients who received craniotomy were enrolled in this study. We found MECA‐79‐positive HEVs in 13 (31%) cases. HEVs density range from 0.01/mm^2^ to 21.13/mm^2^ with a median value of 0.20/mm^2^. We failed to observe any HEVs in 29 cases, mainly due to the small sizes of the resected tumor specimens. Six cases with HEVs density less than 0.10/mm^2^ only showed few locally presented MECA‐79‐positive vessels. These 35 cases were designated as HEV‐Low germinomas. HEVs density in the rest of the seven cases ranged from 0.20/mm^2^ to 21.13/mm^2^. HEVs density of these cases was higher than the median value, so they were designated as HEV‐high germinomas. The grouping was in line with the cutoff point for HEVs density previously described in several kinds of tumors.^12^ The histological morphology of HEVs is shown in Figure 1. The endothelial cells of HEVs possess a cuboidal appearance (Figure 1A–E), while those of ordinary vessels were flat (Figure 1F). Figure 1 also showed the representative images of lymphocytes' extravasation process across the HEVs (Figure 1A–D). The lymphocytes were arrested on the luminal surface of cuboidal endothelial cells (Figure 1A), got in the endothelial lining of HEVs (Figure 1B), then arrived at the external side of endothelial cells (Figure 1C), and finally crossed the basal lamina (Figure 1D). Indented nuclei of endothelial cells, which adjoined lymphocytes were observed in Figure 1B,C. Representative images of immunohistochemical and immunofluorescence staining were shown in Figure 2.

### 3.2 Association of HEVs density with immune infiltration and germinoma cell content

**FIGURE 3:**
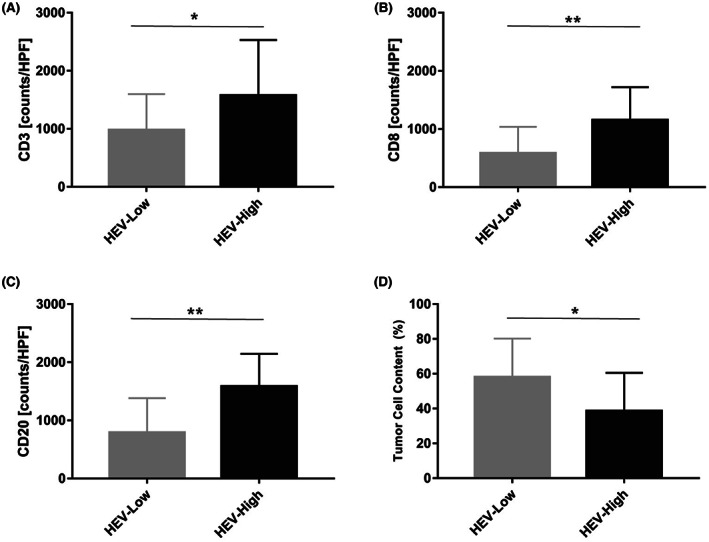
Germinomas with higher HEVs density exhibit higher levels of infiltrating lymphocytes, including CD3 + T cells (A), CD8+ T cells (B), and CD20+ B cells (C). In contrast, tumor cell content was higher in HEV‐low cases (D). *p < 0.05; **p < 0.01.

To investigate the role of HEVs in the process of lymphocyte trafficking in germinoma, we analyzed the association of HEVs density with immune infiltration. The mean amount of CD3+ T cells, CD20 + B cells, and CD8+ CTLs in all the germinomas in this study were 1077.6/HPF, 923.4/HPF, and 678.3/HPF, respectively. The amount of CD3+ T cells in HEV‐high tumors (mean value: 1574.0/HPF) was higher than that in HEV‐low tumors (mean value: 978.3/HPF). The difference was statistically significant (Figure 3A, *p* = 0.041). The same situation existed for CD8+ cytotoxic T cells (mean value 1151.0/HPF in HEV‐high tumors, and 583.7/HPF in HEV‐low tumors, respectively. Figure 3B, *p* = 0.0061). Furthermore, HEV‐high germinomas contain more abundant CD20+ B cells than HEV‐low germinomas (mean value 1584.0/HPF in HEV‐high tumors, and 791.4/HPF in HEV‐low tumors, respectively. Figure 3C, *p* = 0.0023). Representative images of immunohistochemical staining for lymphocytes are shown in Figure 2.

Infiltrating lymphocytes have been proved to be directly cytotoxic to tumor cells in germinoma,^2^, ^3^, ^4^, ^22^, ^23^, ^24^ so we investigated the proportion of neoplastic elements in germinomas. The median tumor cell content was 40% in the HEV‐high group and 60% in the HEV‐low group. As expected, tumor cell content was lower in HEV‐high tumors than that in HEV‐low tumors (Figure 2). The difference between them was statistically significant (Figure 3D, *p* = 0.0428).

### 3.3 Correlation of HEVs density with clinicopathological characteristics

**FIGURE 4:**
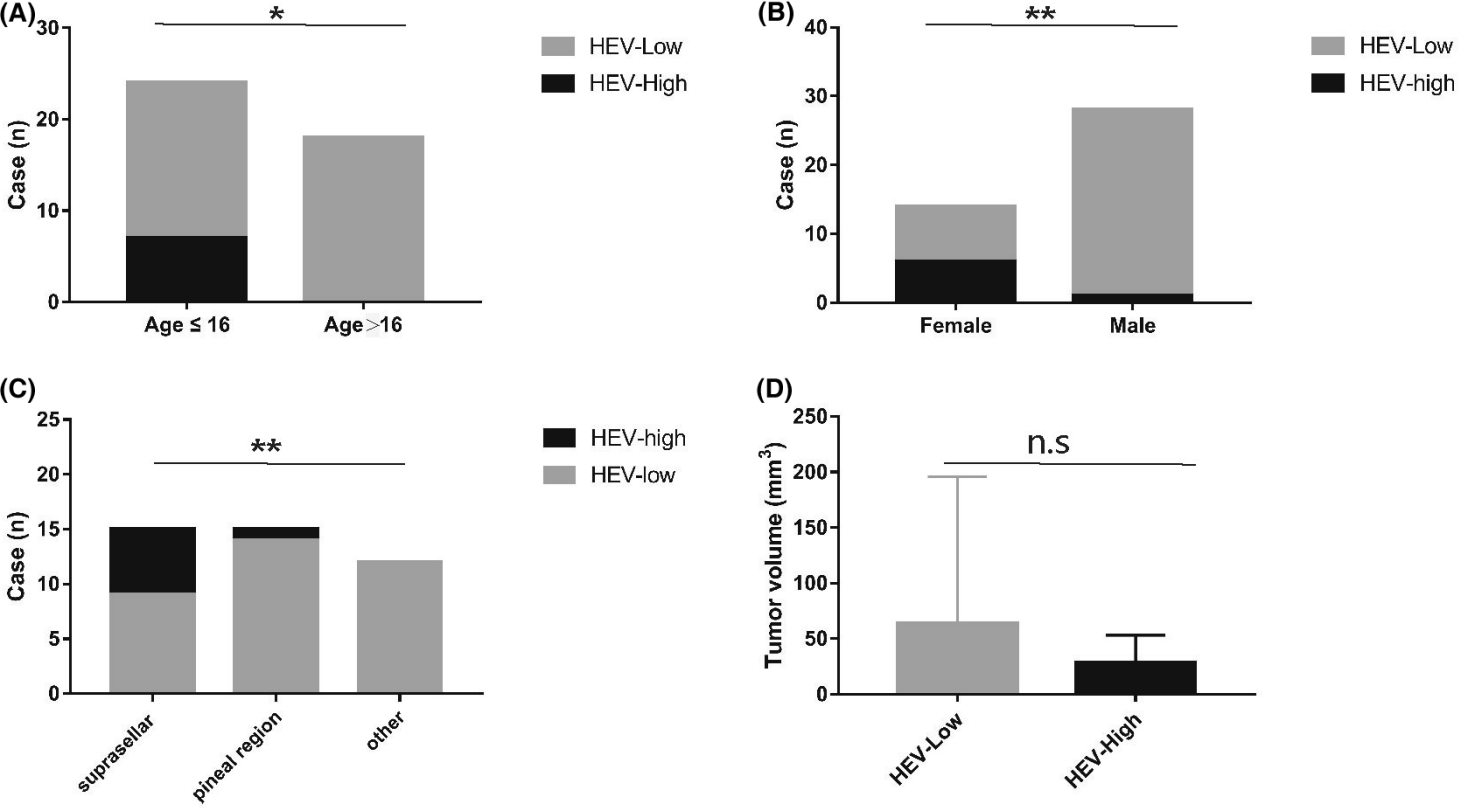
Higher HEVs density was more common in younger (A), female patients (B), and suprasellar germinomas (C). Differences in tumor volume between HEV‐high and HEV‐low cases did not reach statistical significance (D). *p < 0.05; **p < 0.01; N.S:*p* > 0.05.

Clinicopathological characteristics of 42 germinoma patients are presented in Table 1. The median age at diagnosis in our cohort was 16 years. All HEV‐high germinomas were younger than 16 years (Figure 4A, *p* = 0.0121). The age of HEV‐low germinoma patients was evenly balanced (48.6% younger than 16, 51.4% older than 16). In other words, 29.2% (7/24) of the younger cases were HEV high, while none of the older cases were HEV high.

There were 14 female patients and 28 male patients in the present study. We observed significantly higher HEVs density in female patients (Figure 4B, *p* = 0.0013): Six out of 14 (42.8%) female patients were HEV high, whereas only one out of 28 male patients (3.6%) were HEV high.

Besides, HEVs density differed significantly between different tumor locations (Figure 4C, *p* = 0.0093): the proportion of HEV‐high tumors was 40% in germinomas in the suprasellar compartment, 7% in germinomas in the pineal region, and 0% in germinomas in other locations, respectively.

We also compared the tumor volume in HEV‐high and HEV‐low germinomas. The mean value of tumor volume of all germinomas was 57 cm^3^. The volume of HEV‐low tumors (mean value: 63.5 cm^3^) was potentially higher than that of HEV‐high tumors (mean value: 27.9 cm^3^), even though the difference did not reach statistical significance (Figure 4D).

### 3.4 HEV‐related biological processes and enriched cell subsets

**FIGURE 5:**
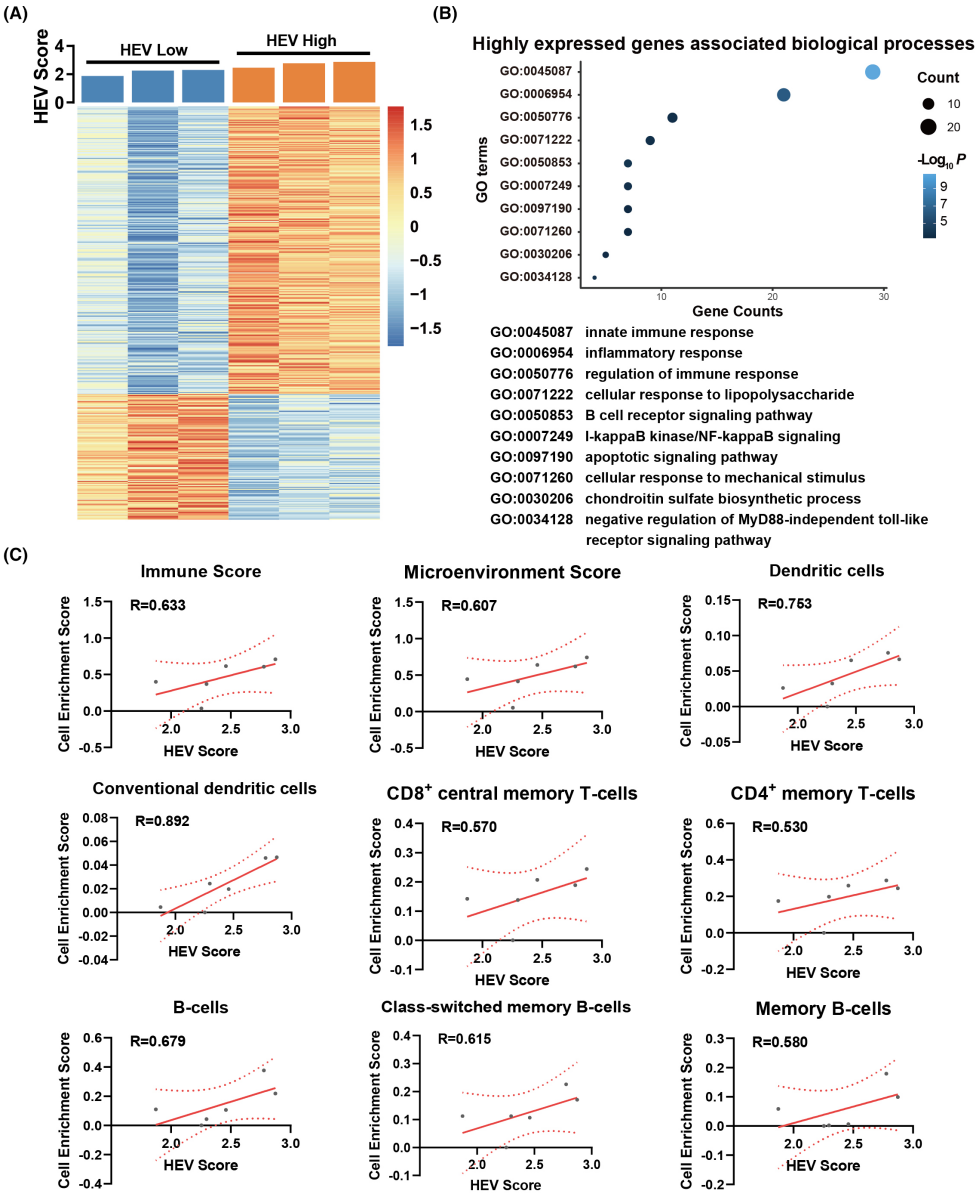
Bioinformatic analysis on intracranial germinomas. The six germinomas were designated as the HEV‐high or HEV‐low groups according to their HEVs enrichment scores. (A) Differentially expressed genes between the HEV‐LOW and HEV‐high groups. (B) GO analysis showed biological processes that HEV‐associated genes mainly enriched in. (C) Correlation analysis between HEV enrichment scores and cell‐type enrichment scores using xCell.

In total, 418 genes were differentially expressed in the HEV‐low and HEV‐high groups, of which 291 genes were highly expressed in HEV‐high germinomas (Figure 5A). Notably, the biological processes analysis showed that HEV‐associated genes mainly enriched in immune‐related GO terms, including innate immune response (GO0045087), inflammatory response (GO0006954), and B cell receptor signaling pathway (GO0050853) (Figure 5B). The xCell analysis revealed germinomas with higher HEVs enrichment scores had significantly increased levels of the immune score, microenvironment score, dendritic cells, CD8+ central memory T cells, CD4+ memory T cells, and B cells (*R* value > 0.5) (Figure 5C).

## 4 DISCUSSION

Germinoma is an intracranial tumor with abundant lymphocytes. However, the mechanisms managing the recruitment of lymphocytes remains poorly understood. In this study, we demonstrated the presence of HEVs in germinoma for the first time. We found that HEVs density was correlated with infiltrating immune cells, including CD3+ T cells, CD8+ cytotoxic T cells, and CD20+ B cells in this intracranial tumor. We also found HEVs were associated with smaller tumor volume and less content of malignant cells in germinoma. Furthermore, bioinformatics analysis showed HEVs‐associated genes mainly enriched in immune‐related biological processes and cell subsets.

Light and electron microscopic examination revealed a plump or cuboidal appearance of the endothelial cells in these HEVs, unlike the flat appearance of endothelial cells in other postcapillary venules (Figure 1). Besides, we also observed lymphocytes' extravasation process at successive stages in different slides. In addition to labeling HEVs and lymphocytes using immunohistochemistry, we also performed immunofluorescence staining to illustrate the spatial relationship between them (Figure 2). In line with a previous study in seminoma,^17^ MECA‐79‐positive HEVs mainly located within the lymphocyte‐rich region in germinoma. Together, these findings proved the presence of HEVs in intracranial germinoma.

MECA‐79‐positive HEVs were observed in the major proportion of several distinct tumor types.^11^, ^12^, ^17^, ^25^ But we only found MECA‐79‐positive HEVs in 31% (13/42) germinomas in the present study. Germinomas are now mainly diagnosed by specimens from stereotactic biopsy other than from surgical resection, so the tissue sample for testing is usually very limited in size. It is plausible to speculate that, cases that showed negative MECA‐79 staining in our study may possess few HEVs elsewhere in the tumor. Some other germinomas showed few focally MECA‐79‐positive HEVs, with the density of HEVs less than 0.1/mm^3^. Together with MECA‐79 negative cases, these cases were classified into the HEV‐Low group, according to the cutoff point defined by a previous study conducted on HEVs in multiple human malignancies.^12^

To clarify whether HEVs play a role in lymphocyte recruitment in germinomas, the association of HEVs density with the level of infiltrating lymphocytes was tested (Figure 3). We found that the amount of infiltrating CD3+ T cells, CD8+ T cells, and CD20+ B cells in the HEV‐high group was higher than that in the HEV‐low group. Based on correlations between infiltrating lymphocytes and HEVs in several other human malignancies,^12^, ^14^, ^16^, ^26^ HEVs have been proposed as the entry point for lymphocyte trafficking in tumors. Our findings suggest that HEVs also play an essential role in lymphocyte infiltration in germinomas.

Hence, HEVs may be essential for effective antitumor immune responses in germinoma and improve patient outcomes. Due to the excellent long‐term survival of pure germinoma, all patients remained disease‐free until the last follow‐up (data not shown), making it difficult to evaluate the correlation between HEVs density and outcomes. That would be the limitation of this study. However, several factors have been shown to possess prognosis significance for germinoma in previous studies. Lower tumor cell content, typical location (neurohypophysis or pineal gland), and abundant infiltrating lymphocytes are significantly associated with a better prognosis of germinoma.^2^, ^27^, ^28^ Interestingly, in the present study (Figure 4), HEVs density was significantly correlated with lower tumor cell content, not to mention their relationship with the amounts of T and B cells. Furthermore, our study also showed that all the HEV‐high tumors were located in the suprasellar and pineal regions. Based on these results, we speculate that HEVs may serve as a predictor of better prognosis in germinomas, yet further studies containing clinical outcome data are required.

Interestingly, we found that most of the cases in the HEV‐high group were female (Figure 4B). It has been reported that males took up the majority of pineal germinomas, while females prevailed in suprasellar ones.^29^, ^30^ In addition, the present study found more HEV‐high cases in suprasellar than in pineal germinomas. Thus, it is possible that the disparity of HEVs distribution between genders is related to different tumor locations in male and female patients.

Bioinformatic analysis showed that HEV‐associated genes were mainly involved in the innate immune response. In accordance with the roles of HEVs in lymphocyte trafficking, HEV‐high germinomas possessed higher levels of the immune score, microenvironment score, B cells, and certain subtypes of T cells. Besides, we observed elevated enrichment scores of dendritic cells and conventional dendritic cells in the HEV‐high group. DCs and DC‐produced lymphotoxin are associated with HEVs formation by downstream LT‐βR signaling in lymph nodes and solid human tumors,^13^, ^31^ which can subsequently activate the NF‐κB pathway to drive the expression of HEV‐associated markers.^32^, ^33^ Indeed, I‐κB kinase/NF‐κB signaling was among the significantly associated biological processes with HEVs in GO analysis in the present study. Altogether, these results reinforced the thesis that HEVs play essential roles in lymphocyte recruitment and immune response in germinomas.

Although malignant, pure germinoma has a significant favorable prognosis due to its sensitivity to radiotherapy and chemotherapy.^34^ However, multiple late adverse effects of these aggressive treatments, such as cognitive dysfunction, growth retardation, and secondary neoplasms, could seriously affect the life quality of young patients with this tumor. Thus, many efforts have been made to find alternative therapeutic strategies to treat this intracranial tumor. Immunotherapy for several kinds of tumors has achieved great success in the last decade. Various types of immunotherapies, such as adoptive lymphocyte transfers, vaccines, and checkpoint blockade, require a sufficient number of inflammatory infiltrations to function as major effector cells for these strategies. Indeed, rich pre‐existing TILS, especially T cells could predict a better response to immunotherapy.^35^ Recently, several studies showed that during immune checkpoint blockade therapy, newly recruited intra‐tumoral T cells from peripheral sites might be associated with clinical response.^36^, ^37^ Furthermore, cytokine LIGHT‐induced HEVs and lymphocyte clusters could sensitize refractory lung metastases to anti‐PD‐1 checkpoint inhibitors.^38^ Florid small lymphocyte infiltration is one of the most striking features of germinoma, which makes immunotherapy a promising treatment for this tumor. Our study showed HEVs might be the significant entry point for lymphocytes to germinoma, and thus may be essential for effective lymphocyte‐based immunotherapies.

## 5 CONCLUSIONS

In summary, this study demonstrated the presence of HEVs in germinoma. Higher HEVs density was correlated with abundant infiltrating lymphocytes and multiple clinicopathological features, which are recognized indicators for favorable prognosis in germinomas. Besides, the bioinformatic analysis showed HEV‐associated genes mainly enriched in immune‐related biological processes and cell subsets. Our findings indicate that HEVs could contribute to lymphocyte recruitment in germinomas, and thus may serve as a predictor of favorable prognosis and better response to immunotherapy in this intracranial tumor.

## AUTHOR CONTRIBUTIONS

**Huiyuan Chen:** Conceptualization (lead); formal analysis (equal); investigation (lead); project administration (equal); software (equal); validation (equal); visualization (lead); writing – original draft (lead); writing – review and editing (equal). **Guilin Li:** Funding acquisition (equal); methodology (equal); resources (equal); supervision (equal). **Yun Cui:** Investigation (equal); methodology (equal); resources (equal). **Qi Zhang:** Methodology (equal); resources (equal); visualization (equal). **Bo Li:** Data curation (equal); supervision (equal). **Xing Liu:** Conceptualization (equal); data curation (equal); funding acquisition (equal); investigation (equal); software (equal); supervision (equal); validation (equal); writing – review and editing (lead).

## CONFLICT OF INTEREST

The authors declare that they have no conflict of interest.

## ETHICS APPROVAL

The study protocol was approved by the Institutional Review Board and Ethics Committee of Beijing Tiantan Hospital.

## PATIENT CONSENT STATEMENT

Written informed consent was obtained from all patients.

## Supporting information


Appendix S1

## Data Availability

The datasets analyzed during the current study are available from the corresponding author upon reasonable request.
